# *Toxoplasma gondii* Oral Infection Induces Intestinal Inflammation and Retinochoroiditis in Mice Genetically Selected for Immune Oral Tolerance Resistance

**DOI:** 10.1371/journal.pone.0113374

**Published:** 2014-12-01

**Authors:** Raul Ramos Furtado Dias, Eulógio Carlos Queiroz de Carvalho, Carla Cristina da Silva Leite, Roberto Carlos Tedesco, Katia da Silva Calabrese, Antonio Carlos Silva, Renato Augusto DaMatta, Maria de Fatima Sarro-Silva

**Affiliations:** 1 Laboratório de Biologia Celular e Tecidual, Universidade Estadual do Norte Fluminense Darcy Ribeiro (UENF), 28013-602, Campos dos Goytacazes, RJ, Brazil; 2 Laboratório de Imunomodulação e Protozoologia, Instituto Oswaldo Cruz (IOC), Fundação Oswaldo Cruz (FIOCRUZ), 21045-900, Rio de Janeiro, RJ, Brazil; 3 Laboratório de Sanidade Animal, UENF, 28013-602, Campos dos Goytacazes, RJ, Brazil; 4 Disciplina de Anatomia Topográfica e Descritiva, Universidade Federal de São Paulo, 04023-900, São Paulo, SP, Brazil; 5 Laboratório de Imunobiologia, Universidade do Estado do Rio de Janeiro (UERJ), 20550-900, Rio de Janeiro, RJ, Brazil; University at Buffalo, United States of America

## Abstract

Toxoplasmosis is a worldwide disease with most of the infections originating through the oral route and generates various pathological manifestations, ranging from meningoencephalitis to retinochoroiditis and inflammatory bowel disease. Animal models for these pathologies are scarce and have limitations. We evaluated the outcome of *Toxoplasma gondii* oral infection with 50 or 100 cysts of the ME-49 strain in two lines of mice with extreme phenotypes of susceptibility (TS) or resistance (TR) to immune oral tolerance. Therefore, the aim of this study was to evaluate the behaviour of TS and TR mice, orally infected by *T. gondii,* and determine its value as a model for inflammatory diseases study. Mortality during the acute stage of the infection for TR was 50% for both dosages, while 10 and 40% of the TS died after infection with these respective dosages. In the chronic stage, the remaining TS succumbed while TR survived for 90 days. The TS displayed higher parasite load with lower intestinal inflammation and cellular proliferation, notwithstanding myocarditis, pneumonitis and meningoencephalitis. TR presented massive necrosis of villi and crypt, comparable to inflammatory bowel disease, with infiltration of lymphoid cells in the lamina propria of the intestines. Also, TR mice infected with 100 cysts presented intense cellular infiltrate within the photoreceptor layer of the eyes, changes in disposition and morphology of the retina cell layers and retinochoroiditis. During the infection, high levels of IL-6 were detected in the serum of TS mice and TR mice presented high amounts of IFN-γ and TNF-α. Both mice lineages developed different disease outcomes, but it is emphasized that TR and TS mice presented acute and chronic stages of the infection, demonstrating that the two lineages offer an attractive model for studying toxoplasmosis.

## Introduction

Infection with *Toxoplasma gondii* is acquired by direct contact with food through ingestion of undercooked or raw meat containing the parasite cysts, congenitally through the placenta [1], or from oocyst contamination of soil or water [2]. In natural oral infections, histopathological studies demonstrate parasite invasion of a variety of cell types in the gut and subsequently disseminating throughout the body [3], [4]. After crossing the intestinal epithelium, *T. gondii* spreads into several tissues and traverses biological barriers to reach immunologically privileged sites such as the brain and eyes where it can cause severe pathologies [5].

A variety of pathological manifestations is observed in *T. gondii* infected murine models, ranging from meningoencephalitis to retinochoroiditis and inflammatory bowel disease (IBD) like [6], [7], [8]. Murine *T. gondii* oral infection is under polygenic control [9], [10]. For some pathology, animal models are scarce and limited for biological reasons. In C57BL/6 mice infected with high *T. gondii* burdens, IBD – like is observed with similar morphopathologic characteristics of human IBD [7]. This inflammatory process results in early mortality of the susceptible hosts. One difficulty in intestinal inflammation studies in mice infected with high *T. gondii* inoculum is maintaining the animal alive during the later stages of the infection. The C57BL/6 develops inflammation and starts to die on the seventh day after infection. Moreover, BALB/c mice survive oral infection with high inoculums but do not develop intestinal inflammation [11]. A more sophisticated model such as TLR4-deficient mice (C57BL/10ScN, carrying a deletion of the TLR4) presents 60% survival after oral infection but reduced immunopathology [12]. This murine ileal immunopathology resembling acute episodes in human IBD [7], [13] suggests that *T. gondii* is involved in the etiopathogeny, especially in human Crohn's disease [14]. Intestinal chronic pathology investigation in mice orally infected with *T. gondii* is therefore relevant.

The role of the commensal intestinal microbiota in colitis has been studied in a number of experimental models, but detailed knowledge on the gut microbiota composition in acute intestinal inflammation is still limited [15]. According to Liesenfeld [7], mice orally infected with 100 cysts of *T. gondii* and treated with ciprofloxacin and metronidazole starting on the day of infection did not develop pathologic changes in their ilea 7 days after infection. The author also states that markedly reduced numbers of intestinal aerobic and anaerobic microorganisms and a shift from gram-negative toward gram-positive organisms were observed in treated mice, pointing toward a role of resident enteric bacteria in the development of intestinal pathology following oral infection with *T. gondii*. Thus it is relevant to probe into the intestinal microbiota in *T. gondii* oral infected mice to better understand the gut inflammatory response.

*Toxoplasma gondii* is the most common cause of retinochoroiditis worldwide in humans representing 28 to 55% of all the posterior uveitis cases [16]. Retinochoroiditis development was detected in histopathological analyses of *T. gondii* infected mice [17], [18]. Tissue cysts have been found in parts of the normal retina surrounding necrotic areas, and cyst rupture may lead to reinfection in addition to subsequent destruction of retinal cells [19]. These observations are mainly in mice infected intraperitoneally [17], inoculated intravitreally or by instillation [20], or congenitally via a mother whose primary infection is acquired during gestation [21], [22]. Although retinochorioiditis develops following oral ingestion in the Syrian Golden Hamster model [23], difficulties are encountered in obtaining an experimental murine model for the study of uveitis caused by *T. gondii* ingestion.

In this study, two lines of mice genetically selected for extreme phenotypes of oral tolerance [24] were infected with *T. gondii* for comparative inter-lineage study. Oral tolerance is a phenomenon that refers to the observation that the ingestion of a protein induces a state of systemic hyporesponsiveness to parenteral immunization with the previously ingested antigen [25]. From the genetic point of view, the oral tolerance character is under polygenic control, and the resistant (TR mice) and susceptible (TS mice) lines are genetically homogeneous at the relevant loci for the selected character and heterogeneous in terms of background genes [24]. As far as the immunological perspective is concerned, TR mice are characterized by the development of strong Th1 and Th2 immune responses and higher acute inflammatory and allergic responses with low CD4+Foxp3+ Treg cell frequency producing insufficient IL-10 levels, in contrast to TS mice [26], [27], [28]. In previous studies, the TR and TS mice proved to be good models when infected with intracellular parasites. *Sporothrix schenckii* and *Leishmania amazonensis* infected TR mice presented, respectively, greater mortality and inflammatory lesions than TS mice due to a more effective immune response [29], [30]. Another reason for adopting these mice is that the involvement of the mucosa with the myriad of antigens from microbial and food proteins may lead to immunogenic and tolerogenic responses [31]. Mucous membranes in general, the gastrointestinal mucosa in particular, are natural and effective ways for *T. gondii* to reach the immune system and disseminate throughout the host.

Considering the divergent TR and TS inflammatory and immuno-tolerogenic profiles produced by genetic selection for extreme phenotypes of oral tolerance, and for their influence on infections, we aimed to know if TR and TS mice orally infected by *T. gondii* may be useful models to study inflammatory diseases caused by this parasite.

## Materials and Methods

### Mice

For each experimental infection, 6 male C57BL/6 mice (5–6 week old) raised at the *Universidade Estadual do Norte Fluminense* and 46 male and female tolerance resistant (TR) and tolerance susceptible (TS) mice (same age) from the *Universidade do Estado do Rio de Janeiro* were used. No sex susceptible difference after *T. gondii* infection was observed between these mice lines. The original population, from which the TR and TS strains were derived, consisted of an equipoise intercross of eight different inbred mouse strains (A/J, DBA/2J, P/J, SWR/J, SJL/J, CBA/J, BALB/cJ, and C57BL/6J) [24]. The consequence of these crosses (F_0_ population) was engaged to initiate bidirectional selective breeding (by assortative mating), developed during 20 consecutive generations of treatment with soluble ovalbumin via the oral route and later challenged with intraperitoneal injections of alum plus ovalbumin. These two lines were selected for extreme phenotypes of oral tolerance (resistant – TR - and susceptible – TS) by an ovalbumin-specific IgG antibody assay and were not isogenic [24]. Mice were housed in a conventional animal facility under controlled temperature, receiving food and water ad libitum.

### Ethics statement

This study was carried out in strict accordance with the Brazilian Law #11794/08. The protocol was approved by Ethics Committee on Animal Use (CEUA) of the *Universidade Estadual do Rio de Janeiro*, Brazil, under n°: CEA/243/2008. Mice were euthanized with CO_2_ in strict accordance with the recommendations in the Guide for the Care and Use of Laboratory Animals of the National Institutes of Health.

### ME-49 strain of *T. gondii*

*Toxoplasma gondii* was maintained in a parasite bank at *Universidade Estadual do Norte Fluminense*. Swiss mice were intraperitoneally inoculated with 20 cysts of ME-49 in 0.2 ml of PBS. When the mice were at the chronic stage (3 months), they were euthanized with CO_2_. Subsequently the brains were removed, homogenized in 1 ml of PBS and cysts without staining were morphologically defined and counted under a microscope (40× objective) on three aliquots of 20 µl.

### Experimental infection

Mice were orally infected by gavage with 50 or 100 ME-49 cysts in 0.2 ml of PBS. Control mice received 0.2 ml of PBS without cysts. Experimental groups were separated according to the following: 23 mice of each lineage (TR and TS); 6 of them used for survival evaluation, 12 used for pathological analysis and the remaining 5 used as controls. All of the C57BL/6 mice were used for survival evaluation. Experimental infection was done in triplicate. The mice were observed during an experimental window of 90 days, on a daily basis. Due to the rapid progression of the disease mice died between monitoring sessions, but the ones found in a moribund state during the daily visit were euthanized with CO_2_. At the end of the experimental window, all the remaining mice were euthanized with CO_2_.

### Quantification of parasites on tissue culture

Parasitic burden evaluation was carried out in the liver and brain by a biological method using monkey kidney fibroblasts (VERO cells), modified from Zenner et al. [4], in triplicates. On the 21^st^ day of infection, these organs from 3 TR and TS mice were collected, washed in PBS, weighed and macerated with a glass stick in PBS (2 ml of PBS for each 0.5 gram). The suspension was centrifuged (50 *g*, 10 min), and 200 µl of the supernatant was added to flasks (25 cm^2^) containing a monolayer of VERO cells in 5 ml of DMEM supplemented with 5% of FBS and 1% ciprofloxacin. After 48 h, the supernatants were collected, centrifuged (1000 *g*, 10 min), the pellet re-suspended in 1 ml and parasites counted in a Neubauer chamber. The number of parasites per gram of organ was calculated as follows: parasitic burden  =  parasite number per ml from tissue culture/organ weight (g). For each organ, the parasitic burden was expressed as a mean log value ±SD. The parasite burden was graphically represented on a linear scale versus the number of cysts used for infection (50 or 100 cysts).

### Quantification of brain cysts

Among the mice selected to evaluate the histopathology, unused survivors were chosen for evaluation of quantity and size of the cysts. 10 TR and 7 TS mice were used in total for this evaluation, 5 TR for each infection dosage, 4 TS mice for 50 cysts and 3 TS mice to 100 cysts infection. The number of brain cysts at the chronic phase of infection (90 days) was directly counted without staining. TR and TS mice brains were removed, homogenized in 1 ml of PBS and cysts were morphologically defined and counted under a microscope (40× objective) on three aliquots of 20 µl.

### Immunofluorescence and quantification of parasites

Frozen sections, 6 µm thick, of liver, brain and ileum of mice infected for 14 days were fixed with acetone. Sections were rehydrated with incubation in a blocking solution (3% bovine serum albumin, 0,1% gelatin, 0,05% tween 20, 0.05% azide in distilled water) for 10 min. Sections were incubated with a polyclonal mice anti-*T. gondii* antibody for 60 min in a humid chamber, washed and further incubated with a secondary antibody conjugated with to Alexa 488 (Molecular Probes) for 60 min. Sections were washed and mounted with Prolong Gold containing DAPI (Molecular Probes), and cured for 24 h in the dark. Slides were observed in a Zeiss Axioplan epifluorescence microscope equipped with a HBO 100 W lamp and digital images were captured with the Axiovision software. Images were processed with similar linear adjustments in Photoshop (Adobe). Parasites in the captured images were quantified using the ImageJ free software and ITCN plugin. The counting parameters were the following: Width – 7; MD – 3.5; Threshold 2.5. Parasites were evaluated in 3 images (20× objective) of 2 independent experiments of each organ and means ± standard deviation values per mm^2^ are given.

### *Toxoplasma gondii* quantification by quantitative real time PCR

Ileum sections of 0.5 cm of 3 infected mice at 7, 14 and 21 days post oral infection were collected, 5 µl of extracted tissue DNA (described above) was added to a reaction mixture containing 10 µl of MasterMix Real Time PCR – SYBR Green, 2 µl of primers TOXO-F (5 mM, 5′-TCCCCTCTGCTGGCGAAAAGT-3′) and TOXO-R (5 mM, 5′-AGCGTTCGTGGTCAACTATCGATTG-3′), and 3 µl of ultrapure water in a final volume of 20 µl. PCRs were performed in a StepOnePlus System (Applied Biosystems). Amplification was performed on the 10 minute initial denaturation at 95°C, followed by denaturation at 95°C for 5 s, annealing at 60°C for 10 s and extension at 72°C for 15 s, 40 times. This was followed by melting curve analysis to check for the presence of primer dimmers or other non-specific PCR products. The standard values of amplification were obtained using concentrations of isolated *T. gondii* with 10-fold serial dilutions, ranging from 10^6^ to 1 parasite. This standard was then compared with the resulting fluorescence of the samples to calculate the number of parasites. Results are presented as means ± standard deviation of a representative experimental out of three.

### Histopathology processing

At 7, 14 and 21 days of infection, 3 mice of each lineage were randomly chosen and euthanized with CO_2_. On the 7^th^ day post infection, the ileum, spleen and liver were collected. At 14^th^ and 21^st^ day of infection, in addition to the organs mentioned above, eyes, brain, lungs and heart were also collected. The eyes were fixed in 2.5% glutaraldehyde in PBS for 24 h and sectioned in a sagital plane, dividing the bulb into 2 hemispheres [20]. The other organs were fixed in 10% neutral buffered formaline in PBS for 24 h and processed for histopathology. Organs of infected mice were embedded in paraffin, sliced and stained with Hematoxylin-Eosin. The slides were observed in a Zeiss Axioplan light microscope, and digital images were captured with an AxioCam Mrc5 with the Axiovision system - Zeiss. Histopathological analysis was carried in all of the 3 experimental infections.

### Evaluation of cytokine production

The levels of cytokine production in the serum of non-infected mice or infected for 7, 14 and 21 days were evaluated in triplicates, using two Cyotmetric Bead Array kits from BD Biosciences: *Mouse Inflammation Kit* and *Mouse Th1/Th2 Kit*. The samples were prepared according to manufacturer's protocol. Data analysis was carried with the FCAP Array v2.0 software, by SoftFlow. Levels of cytokine are in pg/ml.

### Molecular characterization of the commensal intestinal microflora of mice

The ileum content of 3 non-infected mice was examined for the type of commensal microbiota using the primers listed at Table 1. Luminal content was removed from the ileum of the mouse, ressuspended in PBS, centrifuged (16.000 *g* for 10 min), pellet ressuspended in lising buffer (Tris 500 mM [pH 9,0], EDTA 20 mM, NaCl 10 mM, SDS 1%) and incubated with proteinase K (5 mg/ml; Sigma-Aldrich) for 2 h at 56°C. Total DNA served as template for PCR amplification of bacterial 16S rRNA from different groups and genus (products size are indicated in Table 1). PCRs were performed in a final volume of 25 µl, consisting of 8.9 µl of ultrapure sterile water, 2 µl of MgCl_2_ (50 mM), 2.5 µl of reaction buffer (100 mM Tris-HCl, KCl 500 mM), 0.5 µl of dNTP mix, 0.5 µl of each primer, and 10 µl of extracted DNA (10 ng/µl). To these mixtures 0.1 µl of TaqDNA polimerase (Boehringer Mannheim) was added. Amplification cycles were performed in a Veriti DNA termocycler (Applied Biosystems) according to Table 2. 10 µl of the PCR amplification product were run in a 1.5% agarose gel, stained with GelRed, and images were captured in a UV transilluminator. Results are a representative independent experiment out of three.

**Table 1:** Genus/groups, primers, size of the fragments and ATCC references of evaluated bacteria in the commensal intestinal microbiota of TR and TS mice.

Bacteria/Groups	Primer (F/R)	Fragments	Reference
*Bacillus* spp	TCGAAATTGAAAGGCGGC GGTGCCAGCTTATTCAAC	411 bp	ATCC 14579
*Bacteroides* (group)	ATAGCCTTTCGAAAGRAAGAT CCAGTATCAACTGCAATTTTA	501 bp	ATCC 8482
*Clostridium* (group)	AAATGACGGTACCTGACTAA CTTTGAGTTTCATTCTTGCGAA	438-441 bp	ATCC 19403
*Enterobacteriaceae* (group)	CAGGTCGTCACGGTAACAAG GTGGTTCAGTTTCAGCATGTAC	512 bp	ATCC 13048
*Lactobacillus acidophilus*	CACTTCGGTGATGACGTTGG CGATGCAGTTCCTCGGTTAAGC	575 bp	ATCC 4356
*Prevotella* spp	CACRGTAAACGATGGATGCC GGTCGGGTTGCAGACC	527-529 bp	ATCC 49046
*Streptococcus* spp	AGAGTTTGATCCTGGCTCAG GTACCGTCACAGTATGAACTTTCC	500 bp	ATCC 25175

**Table 2:** Genus/groups, numer of cycles, and specific temperatures used to evaluated bacterial in the commensal intestinal microbiota of TR and TS mice.

Bacteria/Groups	Cycles	Denaturation	Anneling	Extension
*Bacillus* spp	35	Initial: 5 min	55°C/1 min	72°C/1 min Final: 4 min
		94°C/1 min		
*Bacteroides* (grupo)	40	Initial: 5 min	50°C/20 s	72°C/30 s Final: 5 min
		94°C/20 s		
*Clostridium* (grupo)	40	Initial: 5 min	50°C/20 s	72°C/30 s Final: 5 min
		94°C/20 s		
*Enterobacteriaceae* (grupo)	35	Initial: 5 min	60°C/30 s	72°C/1 min Final: 10 min
		94°C/30 s		
*Lactobacillus acidophilus*	30	Initial: 5 min	63°C/30 s	72°C/30 s Final: 5 min
		95°C/30 s		
*Prevotella* spp	40	Initial: 5 min	55°C/20 s	72°C/30 s Final: 5 min
		94°C/20 s		
*Streptococcus* spp	30	Initial: 2 min	55°C/15 s	72°C/45 s Final: 5 min
		94°C/15 s		

### Statistical analysis

Statistical differences between the mice groups were determined by ANOVA and Student's *t* test; groups were considered statistically different if *P*≤0.05.

## Results

### TR mice usually died in the initial phase of the disease and TS in the late phase, but TR that survived did so for 90 days

**Figure 1:**
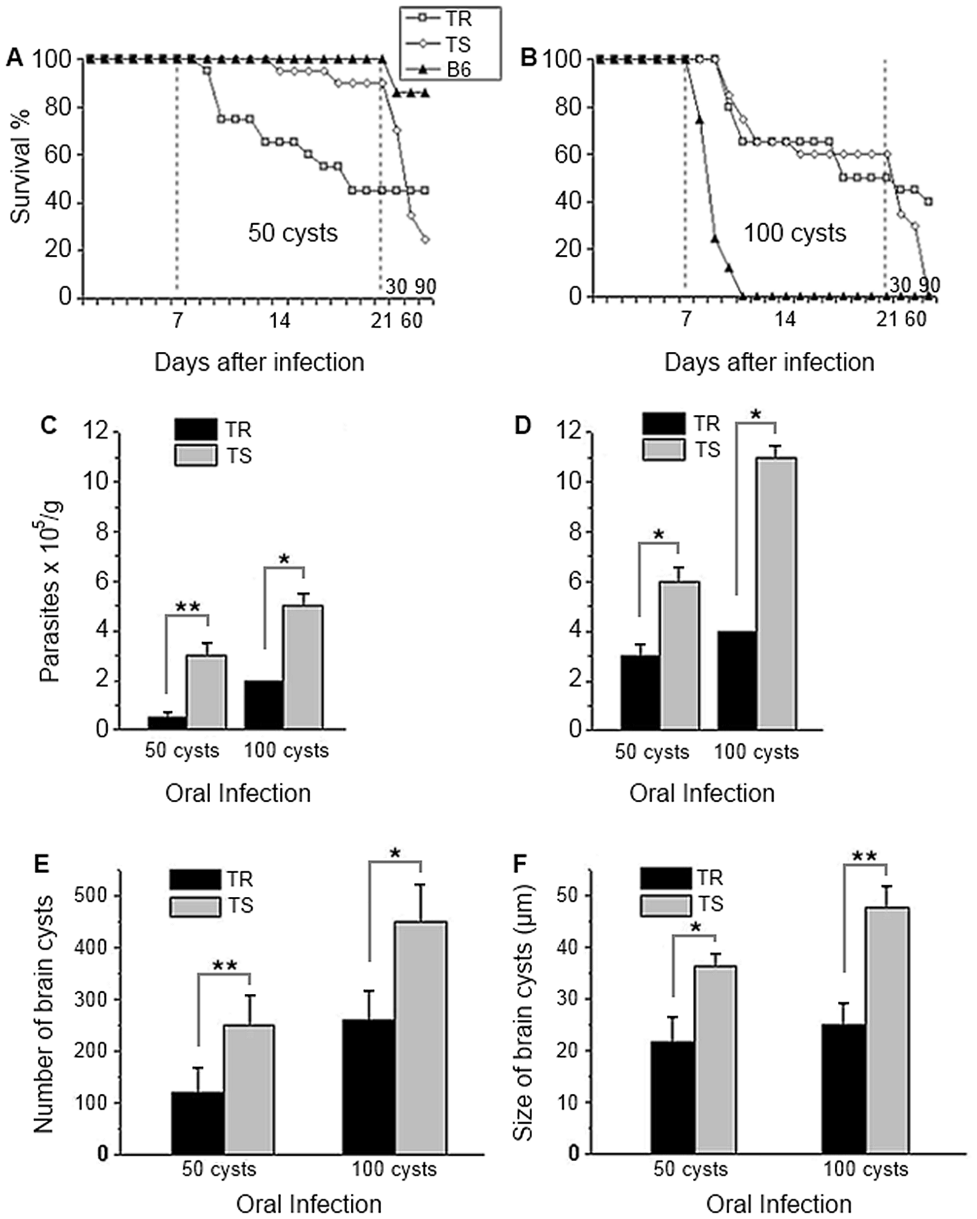
Survival rates of TR, TS and C57BL/6 mice and parasite load on TR and TS after oral infection with *Toxoplasma gondii* cysts. (A–B) The survival was evaluated during 90 days and is presented in percentage. C57BL/6 mice were used for comparative survival study with TR and TS mice. Representative data from three experiments, n = 18 for TR, TS and C57BL/6. (C–D) Number of parasites ×10^5^/g of liver (C) and brain (D) of TR (black bar) and TS (gray bar) mice, 21 days after oral infection with 50 and 100 cysts of *T. gondii*. Data are from 3 independent experiments. Number (E) and size (F) of cysts evaluated in the brains of TR and TS mice at the chronic phase of the infection (90 days) after oral infection with 50 and 100 cysts of *T. gondii*. Means are from six mice per group in two independent experiments. Statistical differences by the Student *t* test are indicated by * (p<0.05) or ** (p<0.01).

When infected with 50 cysts 100% of the C57BL/6 survived after the second week, while TS mice attained 95% survival and TR mice 60% (Figure 1a). In the late phase of the infection, after the third week, the TS mice infected with 50 (Figure 1a) or 100 cysts (Figure 1b) presented high sensitivity with 23 and 0% of survival at the 90^th^ day of infection, respectively. In this same period, there was no mortality of TR mice infected with 50 cysts (Figure 1a) while only 11% of the TR infected with 100 cysts died (Figure 1b).

As expected, C57BL/6 mice did not survive the 2^nd^ week after the initial infection with 100 cysts (Figure 1b). In contrast to the C57BL/6 mice, both TR and TS mice responded similarly to the initial phase of the infection (until the end of the third week) presenting 50 and 60% of survival rate respectively (Figure 1b).

### Lower parasite burden in liver, brain and ileum of TR mice

The parasite burden was quantified in the liver and brain at 21 days of infection. When infected with 50 or 100 cysts, the TR presented significantly fewer parasites in both tissues than TS mice (Figure 1c and d). As expected, infection with 100 cysts resulted in greater parasite numbers than when infected with 50 cysts (Figure 1c and d). Cyst numbers and size were examined at 90 days of infection in the brain of mice. TS mice had more and larger cysts than TR mice regardless of the infection dosage (Figure 1e and f).

**Figure 2:**
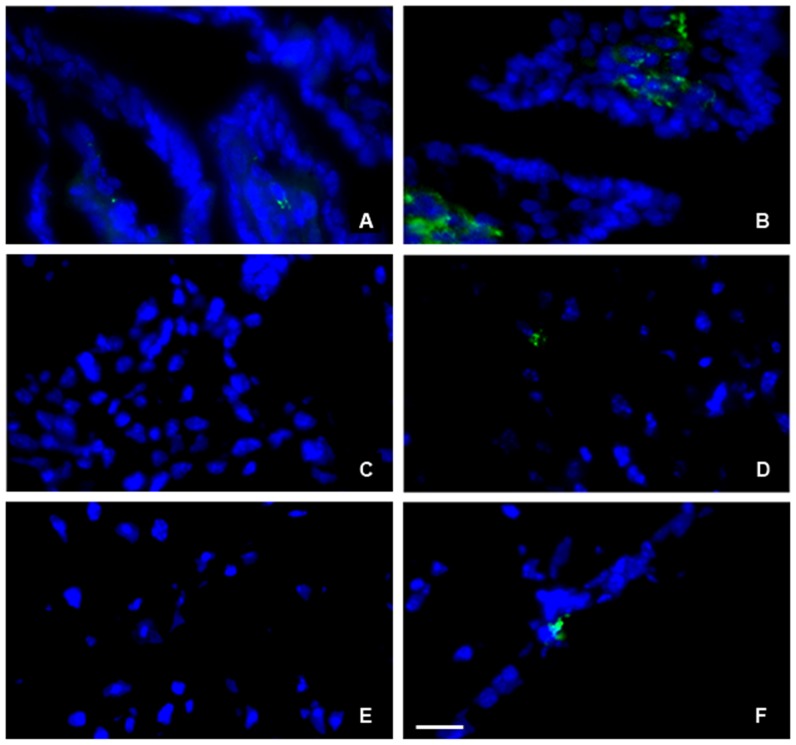
Immunofluorescence analysis of parasite in the ileum, liver and brain of TR and TS mice. Ileum of TR (A) and TS (B) mice. Note higher numbers of parasites in the TS ileum. Liver of TR (C) and TS (D) mice. Brain of TR (E) and TS (F) mice. The cells nuclei were stained by DAPI (blue); Green  =  Anti-*T. gondii*. Bar size: 25 µm.

Immunofluorescence evaluation of parasite burden on ileum, liver and brain of infected mice with 100 cysts was done at 14 days of infection. TR mice displayed lower parasite burden in the ileum in relation to TS mice (Figure 2a and b). Quantification showed a mean of 1580.3±404.35 and 361.3±90.16 parasites per mm^2^ in the ileum of TS and TR mice, respectively. TS mice also presented parasites in the liver and brain (Figure 2d and f) and quantification showed a mean of 160.9±45.63 and 260.8±95.62 parasites per mm^2^, respectively. Liver of TR mice were negative for anti-*T. gondii* and brain had 28.98±40.98 parasites per mm^2^(Figure 2c and e). TR and TS control mice were negative for anti-*T. gondii* (not shown).

**Figure 3:**
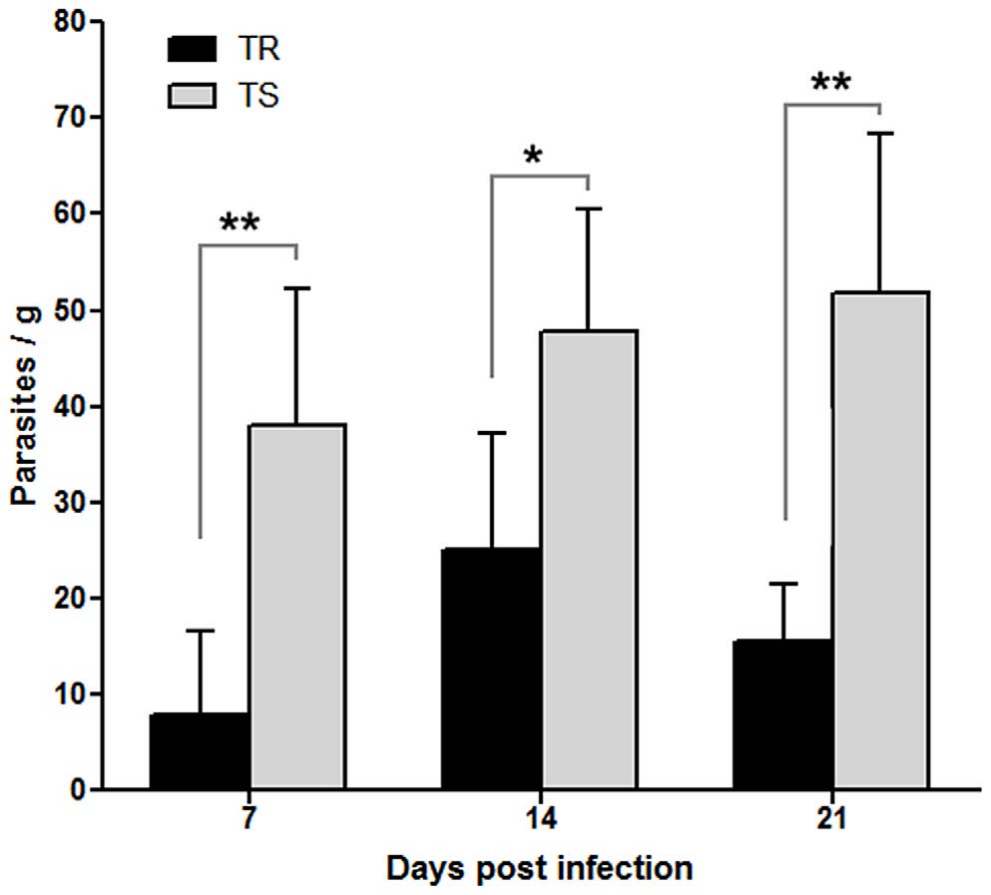
Parasite quantification in ileum of TR and TS mice, infected with 50 cists of ME-49, performed by real-time quantitative PCR. Parasite burden in TR mice (black bar) reached a peak at 14 days of infection and lowered by 21 days, while TS mice (gray bar) showed a constant increase with infection. Values obtained by comparing the amount of fluorescence obtained from serial dilutions, ranging from 1 to 10^6^ parasites. R2 value  = 0.962. Results are from a representative experiment out of three.

Quantitative real time PCR showed that after 7 days, TR mice had about 10 parasites per gram of tissue and TS mice almost five times more (Figure 3). After 14 days of infection, the amount of parasites in TR mice increased in relation to 7 days, as for TS with significant difference between both lineages (Figure 3). After 21 days, the number of parasites decreased in the TR mice, but TS mice showed a constant increase of parasites (Figure 3).

### Severe pathology in ileum, liver and spleen in TR mice in the early phase of infection

**Figure 4:**
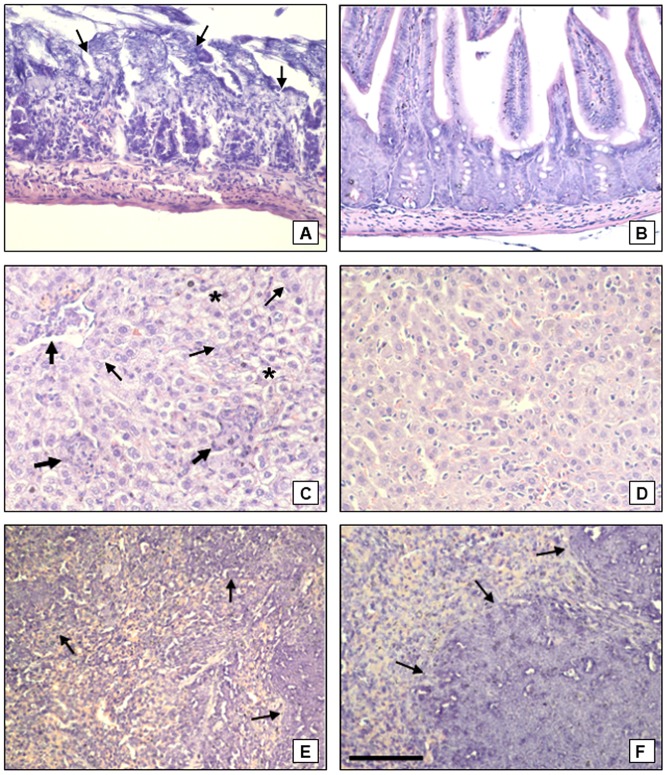
Representative figures of ileum, liver and spleen of TR and TS mice on the 7^th^ day of infection with 50 cysts of *Toxoplasma gondii*. (A) Ileum of TR mice. Severe necrosis of the villi (arrows) can be observed. (B) Ileum of TS mice with normal architecture of the villi and cripts; (C) Liver of TR mice. Severe hydropic degeneration present throughout the tissue (small arrows), associated with focal fatty degeneration (asterisks) and moderate dense granulomas, irregularly distributed (large arrows) can be seen. (D) Liver of TS. The tissues do not display any noticeable alteration. (E) Spleen of TR mice. The spleen shows disorganized germinal centers (arrows) with increased cellularity of the organ at day 7 post-infection. (F) Spleen of TS mice. Organized, well defined germinal centers (arrows), and characteristic lymphoid depletion can be observed at day 7 post-infection. Bar  = 100 µm.

Severe necrosis of ileum villous of TR mice infected with 50 cysts was observed on the seventh day (Figure 4a). Intestinal alterations were not observed in the TS mice (Figure 4b) or in the non-infected control mice (not shown). The TR mice livers presented clear signs of inflammation at the beginning of the infection (7 days) such as numerous granulomas and hydropic degeneration. Focal fatty degeneration was also observed (Figure 4c). TS mice livers did not present granulomas or tissue degeneration at 7 days post-infection (Figure 4d).

The TR mice spleens presented general disorganization of the germinal centers at 7 days post-infection (Figure 4e) that persisted on the examined later time points. TS mice spleens exhibited no change in the organization of the germinal centers during the early phase of the infection (Figure 4f), similar to non-infected TR and TS mice.

### Severe pathology in lung, brain and heart in TS mice in the later phase of infection

**Figure 5:**
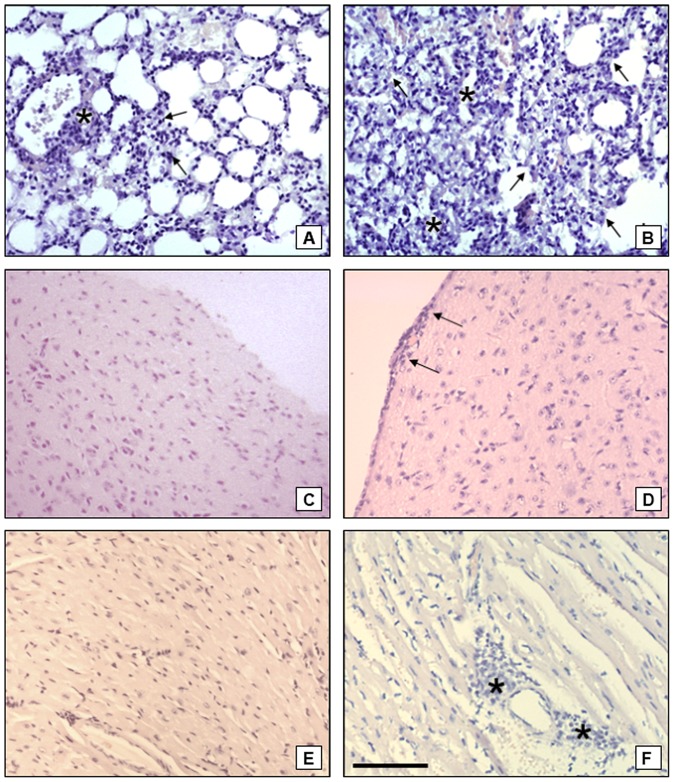
Representative figure of lung, brain and heart of TR and TS mice on the late phase (21^st^ day) of infection with 50 cysts of *Toxoplasma gondii*. (A) Lung of TR mice. Little thickening of the alveolar wall (arrows) can be seen, with presence of few lymphoid infiltrates (asterisk). (B) Lung of TS mice. Areas of inflammation, indicated by the presence of granulomas (asterisks), and thickening of alveolar walls (arrows), caused by the migration of mononuclear cells are observed. (C) Brain of TR mice. There are no alterations throughout the tissue. (D) Brain of TS mice. Increased number of lymphoid cells can be seen in the meninges (arrows), suggesting the development of meningitis. (E) Heart of TR mice. Normal tissue structure can be seen. (F) Heart of TS mice. Mildly dense granulomas (asterisks) can be observed in some areas of the heart. Bar  = 100 µm.

Lungs of TR mice displayed little thickening of the alveolar wall as well as few small lymphoid infiltrates (Figure 5a) compared to non-infected TR mice. Drastic modifications in lungs of TS mice were present in the chronic stage, such as general thickening of the alveolar wall and massive infiltration areas, as observed on the 21^st^ day post-infection (Figure 5b).

TR mice brains presented small mononuclear infiltrates with no change during the infection period (Figure 5c). However, TS mice had mononuclear focal infiltrates, gliosis, perivascular cuffing (not shown) and meningitis (Figure 5d).

The heart evidenced pericarditis and sparse granulomas at 14 days of infection in TR mice (not shown). On the 21^st^ day of infection, the TR mice showed no signs of inflammation (Figure 5e), but the TS mice had lymphoid infiltrates and granulomas of moderate density (Figure 5f).

### Inflammation of the ileum of TR mice caused by the infection resulted in surviving mice

**Figure 6:**
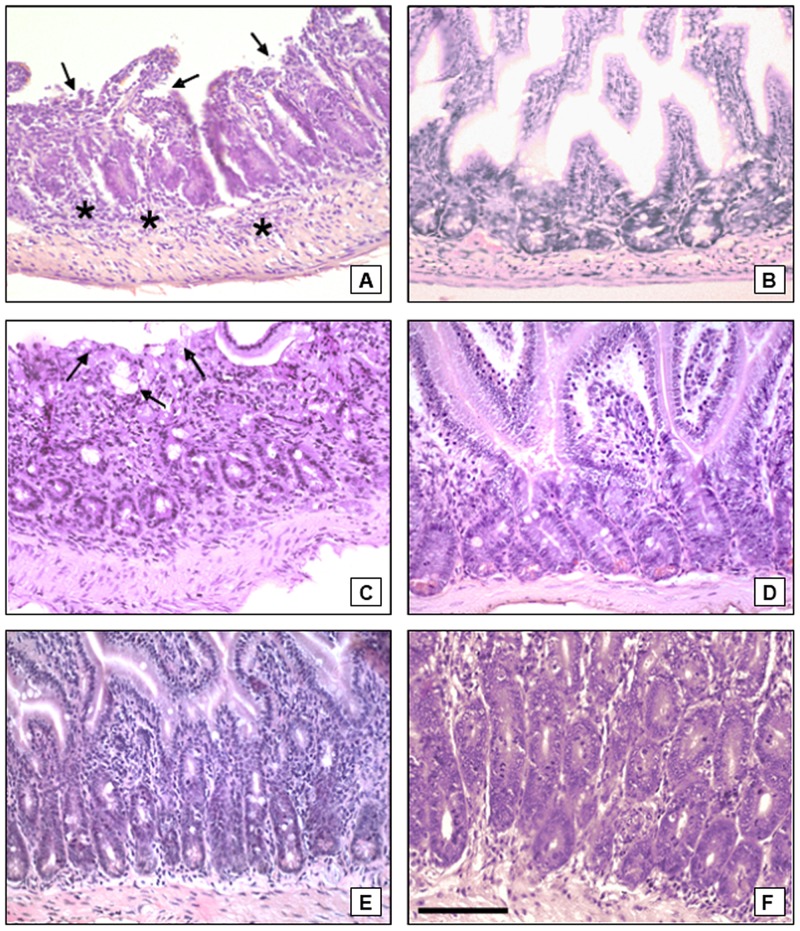
Ileum inflammation kinetic of TR and TS mice caused by *Toxoplasma gondii* 100 cysts infection. (A) TR mice, at 7 days of infection. Infiltration of lymphoid cells in the villi and lamina propria (asterisks) and necrosis of villi (arrows) can be seen. (B) TS mice, 7 days after infection. Preserved villi and lamina propria free of infiltrates are observed. (C) TR mice, 14 days after infection. Occasional areas of necrosis (arrow) are seen. (D) TS mice, at 14 days of infection. Preserved villi and lamina propria free of infiltrates can be observed. (E) TR mice, 21 days after infection. Healthy villi can be observed. (F) TS mice, 21 days after infection. Villi have normal appearance. Bar  = 100 µm.

The TR mice displayed clear signs of ileal necrosis on incipient infection (Figure 6a), evolving to occasional areas of necrosis at day 14 (Figure 6c) and finally disappearing on day 21 (Figure 6e). TS mice had no classical ileal necrosis phenotype (Figure 6b, d and f). However, some mice exhibited small mononuclear infiltrates (not shown). The intestinal inflammation evaluation of TR mice until its resolution was possible by its longer survival, differently from isogenic mice.

### Retinochoroiditis and other disorders in the retina in TR and TS mice at 21 days of infection

**Figure 7:**
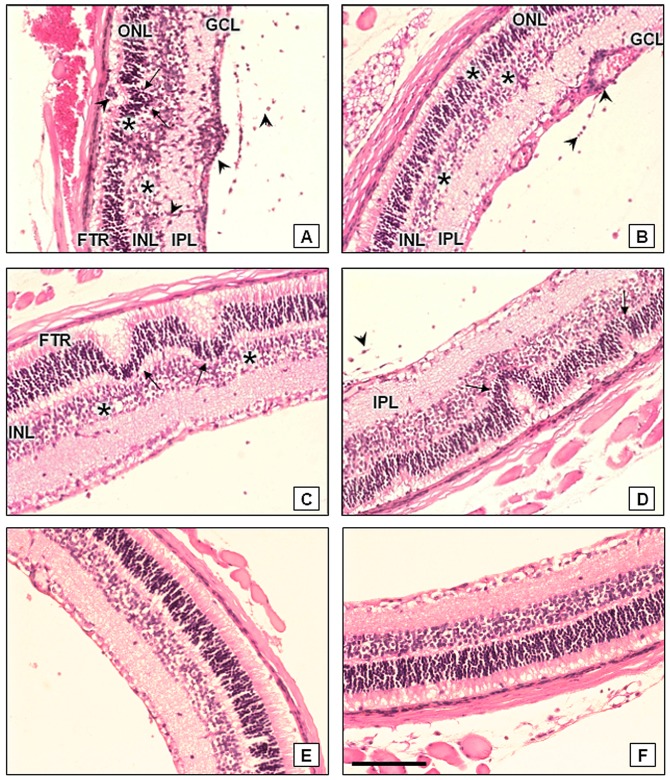
Retinae of TR and TS mice during the course of oral infection with 100 cysts of *Toxoplasma gondii*. (A) TR mice, at 21 days of infection. Note focal retinochoroiditis (arrows), with alterations in the disposition and cytoarchitecture of the layers of the retina; intense inflammatory infiltrate (arrowheads) in the outer segment of photoreceptors (FTR), inner plexiform layer (IPL), ganglion cell layer (GCL), and the vitreous body. Edema (asterisks) were frequently observed in the inner (INL) and outer nuclear layers (ONL). (B) TR mice, at 21 days of infection. Vasculitis (arrowheads) and inflammatory infiltrate in the IPL, GCL and vitreous and edema in ONL and INL (asterisks). (C) TR mice, at 21 days of infection. Multifocal retinochoroiditis (arrows), resulting in complete dissociation of the FTR, and frequent edema (asterisks) in the INL can be observed. (D) TS mice, after 21 days of infection. Retina showing multiple foci of retinochoroiditis (arrows), with mild inflammatory infiltrate in the IPL and in the vitreous (arrowhead). (E) Non infected TR mice. Preserved cytoarchitecture of retina. (F) Non infected TS mice. Absence of alterations in the layers of the retina. Bar  = 100 µm.

Histopathological data revealed that 80% of the TR mice showed ocular inflammatory infiltrates often observed in the inner plexiform layer, ganglion cell layer (Figure 7a) around the vessels and in the vitreous (Figure 7b). The lesions were characteristic of retinochoroiditis with changes in the disposition and morphology of the retina layers, such as the outer segments of the photoreceptors, outer nuclear layer, outer plexiform layer and inner nuclear layer (Figure 7a and c). Only 33% of the TS mice had lesions in the retina, with a mild inflammatory infiltrate in the vitreous and inner plexiform layer (Figure 7d). Noninfected TR (Figure 7e) and TS (Figure 7f) mice display normal retina architecture.

### TR mice showed inflammatory cytokine profile

**Figure 8:**
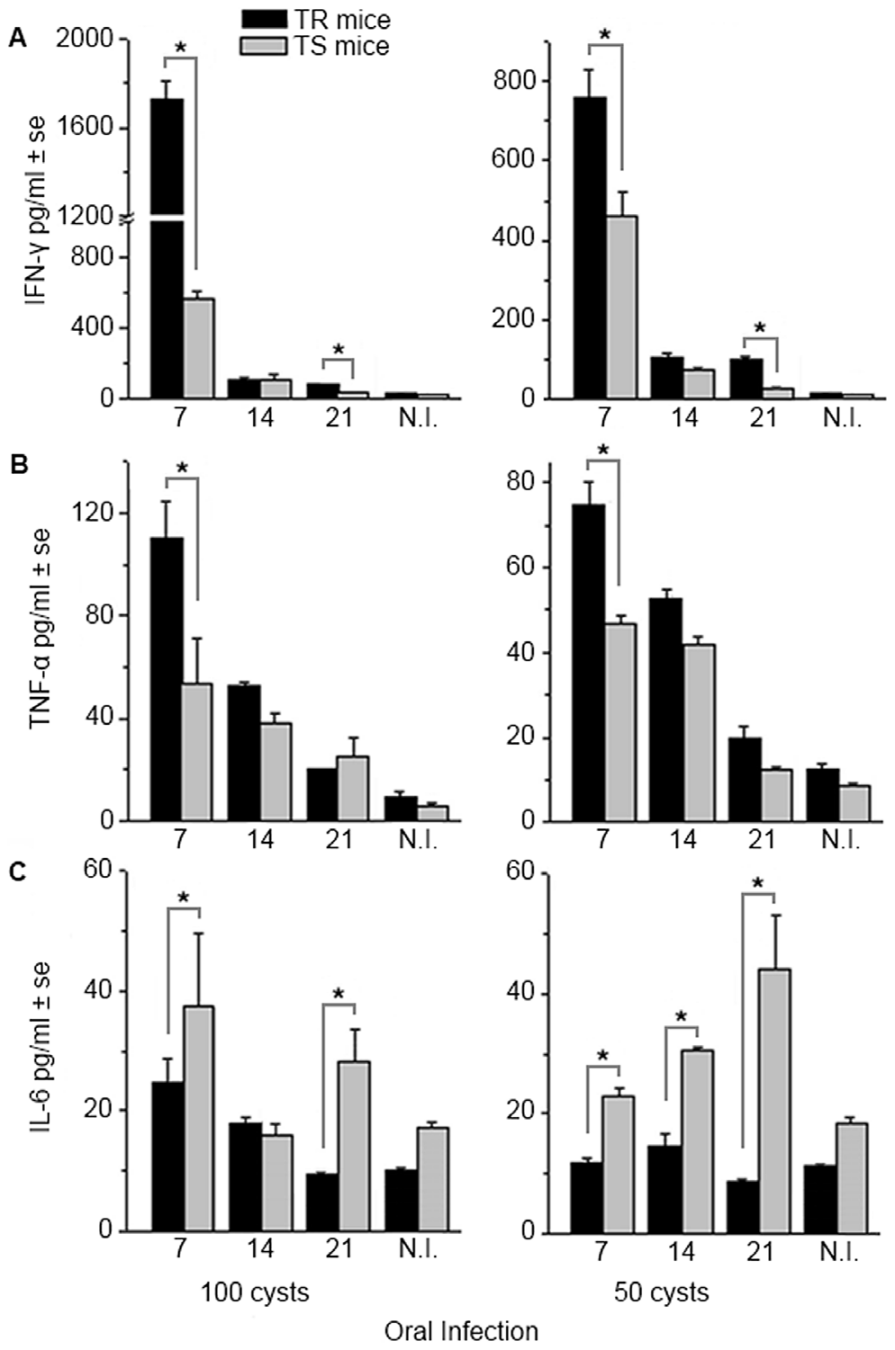
Cytokines concentration in sera (pg/ml) of TR and TS mice measured on days 7, 14 and 21 after the oral infection with 100 and 50 cysts of *Toxoplasma gondii*. (A) Concentration of IFN-γ. The TR mice presented about 3 times higher concentrations than TS mice at 7 days of infection. The titers dropped at 14 and 21 day both in TR and TS mice, but TR mice presented higher values on the 21^st^ day of infection compared with TS mice. (B) Concentration of TNF-α. The titer was higher in the TR mice than in TS mice, especially during the acute stage of toxoplasmosis. Along the course of the disease, concentration of TNF-α in both groups of mice displayed lower titers. (C) Concentration of IL-6. TS mice displayed higher titers of IL-6 during the course of infection compared with the TR mice. Both infected lineages presented higher concentrations, when confronted with non-infected mice. N.I. – non-infected. Bars represent the mean ±SE of three experiments with five mice per group. Statistical differences by the Student *t* test are indicated by * (p<0.05).

IFN-γ production was higher in TR mice at the beginning (7 days) and later (21 days) at the infection independently if the 100 or 50 cysts were used (Figure 8a). TNF-α production had a similar production trend as IFN-γ on the 7^th^ day of infection; no difference between these mice was detected later on during the infection (Figure 8b). Both of these cytokines lowered the levels of production with infection time and 100 cyst infection resulted in higher production levels (Figure 8a and b). When 100 cysts were used to infect mice, IL-6 production was significantly higher in TS mice only at 7 and 21 days of infection (Figure 8c). IL-6 production increased in TS mice with the course of the infection when 50 cysts were used; TR mice presented similar low levels of this cytokine (Figure 8c).

### TR and TS mice presented similar commensal intestinal microbiota profiles

**Figure 9:**
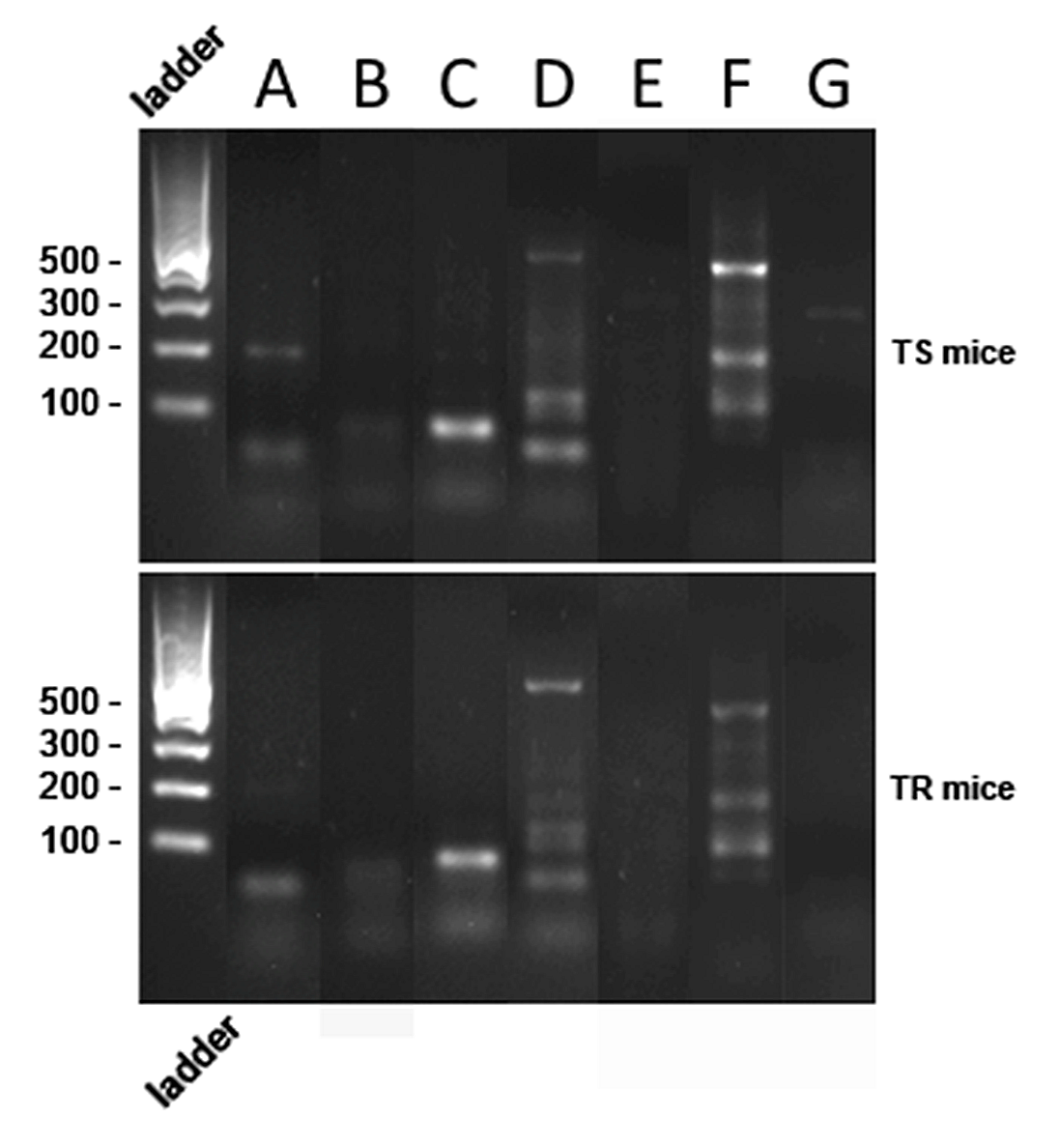
TR and TS mice have similar commensal ileal microbiota evaluated by PCR. (A) *Bacillus* spp.; (B) *Bacteroides* group; (C) *Clostridium* spp.; (D) *Enterobacteriaceae*, PCR product around 500 bp; (E) *Lactobacillus acidophilus*; (F) *Prevotella* spp. PCR product around 500 bp; (G) *Streptococcus* spp. Non-infected TR and TS mice were at the same housing conditions. Bacteria were identified by comparative sequence analyses of 16S rRNA gene fragments amplified from reference species. Gel stained with GelRed, images captured under UV transilluminator. Note similar results for both mice lineages. Results are from a representative experiment out of three.

Because the commensal intestinal microbiota determines the inflammatory result of *T. gondii* oral infection in mice [7], [15], the presence of classical groups and genera of bacteria composed of Gram-positive and Gram-negative, aerobic and anaerobic, were evaluated in TR and TS mice (Table 1). PCR analysis showed that both mice lineages had a similar bacteria profile, mainly composed of *Enterobacteriaceae* and *Prevotella* spp. (Figure 9). The bands observed for *Bacillus* spp., *Bacterioides* and *Clostridium* (Figure 9) are outside the expected amplification size (Table 1) and, thus, are negative. The other genera showed no amplification products (Figure 9).

## Discussion

When infection is through the oral route, *T. gondii* enters the organism by first infecting epithelial cells in the intestine, crossing the basal membrane and migrating within the lamina propria [5]. There, dendritic cells (CD11b^+^ CD11c^+^) and macrophages (CD11b^+^ CD11c^−^) are infected and are probably responsible for the dissemination of the parasite through blood to distant sites [32]. *Toxoplasma gondii* infection can cause different clinical manifestations and the immune response, both locally and systemically, vary among individuals by their diverse genetic backgrounds and immune status [33]. In this study, TS and TR mice were showed to be useful models for toxoplasmosis after oral infection. A summary of the results obtained with both mice lineages are in Table 3. TR mice demonstrated a high mortality rate in the early phase of the infection with both cysts dosage, maintaining about 40% of survival rate at 90 days of infection. TS mice presented lower mortality in the early phase of the infection, but only 23% survival after infection with 50 cysts, and 0% survival with 100 cysts at the 90^th^ day post-infection. During the acute stage, the TR mice carried a lower parasite load in the ileum, liver and brain than TS mice. Extensive ileum necrosis was observed in TR mice, whereas TS mice presented less alteration, characterized as small points of sequelae. Moreover, TR mice had a clear pro-inflammatory cytokine profile based on elevated IFN-γ and TNF-α profiles, and small IL-6 production. Thus, TR mice, due to their high inflammatory response, may die during the early stages of toxoplasmosis from intestinal necrosis. Moreover, TR mice survival at the late phases after infection may be explained by their intense inflammatory response controlling the proliferation of the parasite during the initial stages of infection, allowing the mice to have a milder illness and survive to late periods of infection. This is corroborated by the fact that TR mice possess few CD4^+^ Foxp3^+^ cells, producing low anti-inflammatory cytokines levels which imply a high inflammatory potential [26], [28]. TS mice, on the other hand, carry high numbers of regulatory T cells CD4^+^ Foxp3^+^, thus, better regulating the inflammatory response [26], [28]. The 100% mortality rate of C57BL/6 were expected, occurring after de second week after infection, due to the intense inflammatory responses in this mice line. This characteristic is due to the mouse single genome (no genetic variance), a hindrance for this animal as a model for human disease studies involving a complex genetic and environmental etiology [34], [35].

**Table 3:** Summary of the results obtained with TR and TS mice after infection with *Toxoplasma gondii*.

	TR	TS
**Acute survival**	50 cysts infection, mice presented 61% survival at 14^th^ day, reaching 44% at 21^st^ day. 100 cysts infection, mice presented 61% survival rate at 14^th^ day, and 50% at 21^st^ day.	50 cysts infection 94% of mice survived until 14^th^ day, and 89% survived until 21^st^ day. 100 cysts for infection, mice presented 61% survival at 14^th^ day, and 55% at 21^st^ day.
**Chronic survival**	For 50 cysts infection, 44% of the mice survived until 90^th^ day. Using 100 cysts, mice presented 44% survival at 30^th^ and 60^th^ day, reaching 39% survival at 90^th^ day.	50 cysts infection, 72% survived at 30^th^ day, decreasing to 23% survival at 90^th^ day. 100 cysts infection, 38% of the mice survived at 30^th^ day, and none of them survived at 90^th^ day.
**Parasitic load**	Infected TR mice presented fewer parasites in both liver and brain, when compared to TS mice. Immunofluorescence quantification showed, in a similar manner, less parasites in ileum and brain. Quantification by qPCR demonstrated a small quantity of parasites in ileum at day 7, an increase by day 14, and decreased by day 21.	TS mice presented more parasites in the liver and brain after infection. Immunofluorescence quantification showed four times more parasites in the ileum, more parasites in the brain and presence of parasites in the liver. qPCR quantification showed almost five times more parasites than TR mice, and a continual increase in this number along the infection.
**Inflammation**	The ileum, liver and spleen of infected TR mice displayed signals of acute infection, mainly at 7 and 14 days of infection. In the ileum these inflammation signs reverted back to a normal state by 21 days of infection. Brain, lungs and heart showed little or no significant alterations.	TS mice ileum, spleen and liver did not show signs of inflammation in the 7^th^ day of infection. The ileum kept its normal appearance by 21^st^ day of infection. Lungs, brain and heart, at 21^st^ day, presented signs of inflammation, respectively.
**Retinitis**	33% of the mice presented inflammatory infiltrations in the vitreous and inner plexiforma layer, with edema.	80% of the mice presented alterations in the ocular cytoarchitecture, with vasculitis, edema, inflammatory infiltrates and dissociation of photoreceptor layer.
**Seric IL-6**	Mice infected with 50 cysts had no significant alteration in IL-6 production. Infection with 100 cysts caused an increase in IL-6 production at the 7^th^ day of infection, decreasing by the 14^th^ day with normal level at the 21^st^ day.	At 7^th^ day of infection with 50 cysts IL-6 increased, double the normal production by the 21^st^ day. 100 cysts infection highly increased the levels of IL-6 that decreased to normal level at the 14^th^ day.
**Seric IFN-γ**	IFN-γ was high at the 7^th^ day, decreasing at 14^th^ day and maintaining the same quantity at 21^st^ day post infection. At 7^th^ and 21^st^ days, the levels were higher than in TS mice.	IFN-γ for TS mice increased to near 500pg at day 7 of infection, dropped at day 14, almost to the same as TR mice. In the 21^st^ day, the levels of IFN- γ in TS mice were similar to normal levels.
**Seric TNF-α**	TNF-α at day 7 was high, at 14 days of infection the levels started to decrease and continued at 21^st^ day, although not to normal value.	TNF-α level in TS mice were always lower than in TR mice with similar decreasing behavior with infection.

In the early infection, TR mice displayed intense lesions in the ileum, liver and eyes, as well as disorganization of the germinal centers of the spleen, suggesting a strong immune response in the pathogenesis. This gains further strength after detection of higher levels of pro-inflammatory cytokines such as IFN-γ and TNF-α and lower level of pleiotropic cytokine IL-6 in these mice during the initial stage of infection. C57BL/6 also produce high levels of IFN-γ and TNF-α [7] leading to a similar outcome as TR mice, with notable exception of the eye infection that is not detected in the former mice line. For the TS mice, the production of IFN-γ and TNF-α was lower than for TR mice during the acute stage, and in the early chronic stage these levels were almost the same as non-infected mice, but IL-6 levels were greater. It is known that increased levels of IL-6 antagonize IL-12 and IFN-γ production, resulting in a profound anti-inflammatory signal that blocks the generation of protective Th-1-type immunity to *T. gondii* [36]. Similar results were observed after *L. amazonensis* infection, where TR mice presented intense inflammatory lesions and high cellular immune responses with low parasite load and low anti-inflammatory cytokine levels, in contrast to TS mice which had few inflammatory lesions and low cellular immune responses with high parasite load and high anti-inflammatory cytokine levels [30].

It is known that the composition of the commensal intestinal microflora is important in the inflammatory response outcome in mice after *T. gondii* oral infection [7], [15]. Both mice lineages used in this study presented similar composition of the commensal intestinal microflora. However, distinction on the size of the amplified bands of *Prevotella* spp and *Enterobacteriaceae* was observed between TR and TS mice suggesting that there may be a quantitative difference in these bacterial population. Colonization of mice intestine with *Prevotela copri* increases the sensitivity to chemical induced colitis due to its pro-inflamatory potential [37]. Thus, the inflammatroy differences seen in TR and TS mice may be influenced by quantitative differences of intestinal microflora. Further experiments moduating the commensal intestinal microflora of these mice may be important to further probe into their inflammatory response after *T. gondii* infection.

Gazzinelli et al. [38] reported that IL-10 knockout (KO) mice inoculated with *T. gondii* of the ME-49 strain, succumbed within the first two weeks of the infection, presenting a massive generalized lymphocytic infiltration with extensive hepatic necrosis and no evidence of enhanced parasite proliferation and inflammation in the central nervous system. Half of TR mice died by the third week after oral infection with *T. gondii* exhibiting ileum and hepatic injury despite the lower parasite load. However, after this period there was a recovery and long survival of the remaining TR in contrast to the TS mice that presented massive infiltration of mononuclear cells in the lungs, heart and brain and increased parasite load, as well as larger cysts in the brain. The high survival of TS mice infected with 50 cysts during the initial stage of infection can be explained by low-inflammatory response. Even when infected with 100 cysts, although survival decreased, histopathological data of TS mice indicated low inflammatory response, which allowed the maintenance of tissue integrity. In both cases, higher levels of IL-6 were detected, suggesting that this cytokine plays a fundamental role in this system. Stumhofer et al. [39] identified a new pathway reporting that IL-27 and IL-6 induced T helper type 1 and 2 cells, as well as T helper cells that produce IL-17, to secrete IL-10. Low inflammatory activity of TS can be explained by its high production of IL-6, limiting inflammatory responses by induction of IL-10 production and by its inhibitory effects on IL-12 and IFN-γ[36], [39], [40]. The high levels of IL-6 observed in TS mice infected with 100 cysts could be induced by the elevated number of parasites inoculated, leading to a strong inhibition of Th-1 response, and possible induction of IL-17 production via Th-17 cells, effectively limiting the ability of these mice to control the parasite replication [39]. The elevated levels of this cytokine could also be correlated with the pathology observed in the lungs and brain of the TS mice [36]. It is known that the TS mice submitted to different inflammatory and allergenic stimuli respond with a high level of IL-10 [26], [27], [30]. However, further studies on the evaluation of cytokines such as IL-10, IL-12 and IL-17 becomes necessary for the TR and TS mice.

Classical intestinal necrosis in TR mice infected with 50 or 100 cysts was observed not only early in infection but also at the 21^st^ day when recovery was ensued. Similar intestinal inflammation caused by *T. gondii* oral infection was observed in C57BL/6 mice, but only after infection with high cysts numbers. However, in this later model, no mice survived to the 2^nd^ week, limiting the study of inflammation as previously described by Liesenfeld et al. [41]. In TR mice, however, we observed the recovery of ileum after the second week of infection. This is supported by the reduction of IFN-γ and TNF-α production at the end of the acute (14^th^ days) and beginning of the chronic stage (21^st^ days) of the infection, suggesting a resolution of the inflammatory reaction in TR mice that survived the acute phase of the infection. In humans, IBDs such as ulcerative colitis or Crohn's disease frequently cause epithelial damage in the intestine. In general, the intestinal epithelium is able to rapidly repair itself by restitution, proliferation and differentiation of epithelial cells when such tissue damage occurs [42]. It is also evident that IL-10 produced by T cells can inhibit inflammation in the gut and thus the development of severe enterocolitis [43], [44]. In toxoplasmosis, Tregs are essential during protection in early phase of *T. gondii* infection. Depletion of CD25^+^ led to a rise in mortality in resistant BALB/c mice infected with *T. gondii* [45]. Further experiments are necessary to determine if these cells are involved in the resolution of the inflammation in TR mice that survived the initial phase of the infection.

The immune privilege within the eye is maintained by a combination of anatomical, physiological and immuno-regulatory mechanisms, including immunosuppressive factors such as IL-10, produced by CD4^+^ CD25^+^ T cells [46]. Considering that *T. gondii* infection is acquired through the oral route, potential mechanisms for parasite dissemination from the intestinal lumen to the brain and retina have been considered [19]. However, there is a lack of animal models for ocular toxoplasmosis after oral infection [23]. Our experimental results demonstrate that 80% of the TR mice infected with 100 cysts by the oral route developed lesions characteristic to retinochoroiditis. Merely 33% of the TS mice had lesions in the retina. Thus, TR mice orally infected constitute a suitable model for the study of retinochoroiditis and lesions in the retina. It was described in the literature that differences in gender have been shown to affect susceptibility to *T. gondii* infection in murine models using the intraperitoneal and peroral route of infection [47]. However no sex susceptible difference after *T. gondii* infection was observed in TR or TS model.

## Conclusions

We suggest that the tolerogenic activity of TS mice through the action of anti-inflammatory cytokines by regulatory cells is able to decrease inflammatory immune responses caused by *T. gondii* infection, promoting better survival of mice during the early phase of the infection with 50 cysts, but leading to poor parasite control and an increased number of cysts and growth of parasites in late stages of the infection with both doses of infection. This is further corroborated by the higher production of IL-6 by these mice, which has an indirect anti-inflammatory action against TNF-α and IFN-γ. The high mortality of TR mice in the early phase and low mortality in the late phase, together with the lower number of cysts and parasites, suggest that the higher immune capacity of TR mice enables control of parasite growth already upon incipient infection. Moreover, the immunogenic and inflammatory profile of TR mice may play a crucial role in the lesions, presence of inflammatory infiltrates and granulomas in the ileum, eyes, liver and spleen. In contrast to TS mice, the low level of pleiotropic cytokines like IL-6 (seen here) and regulatory cytokines like IL-10 [30] in TR mice may be pointed as a mechanism responsible for these lesions and render them unable to counteract the inflammatory effect induced by the infection. Hence, the mortality and pathology of orally infected TS and TR mice in this study is in accordance with their oral tolerance profiles provided by several generations of cumulating genes for high and low tolerogenicity, respectively, reinforcing their validity as a model for *T. gondii* infection studies.
